# Mitochondrial DNA diversity of D-loop region in three native Turkish cattle breeds

**DOI:** 10.5194/aab-66-31-2023

**Published:** 2023-01-24

**Authors:** Eymen Demir, Nina Moravčíková, Bahar Argun Karsli, Radovan Kasarda, Ibrahim Aytekin, Umit Bilginer, Taki Karsli

**Affiliations:** 1 Department of Animal Science, Faculty of Agriculture, Akdeniz University, Antalya, 07058, Türkiye; 2 Institute of Nutrition and Genomics, Faculty of Agrobiology and Food Resources, Slovak University of Agriculture in Nitra 94976, Slovak Republic; 3 Department of Agricultural Biotechnology, Faculty of Agriculture, Eskisehir Osmangazi University, Eskisehir 26040, Türkiye; 4 Department of Animal Science, Faculty of Agriculture, University of Selçuk, Konya 42130, Türkiye; 5 Department of Animal Science, Faculty of Agriculture, Eskisehir Osmangazi University, Eskisehir 26160, Türkiye

## Abstract

This study aimed to reveal the genetic variability of mitochondrial DNA (mtDNA) displacement-loop (D-loop) region in 62 animals belonging to three native Turkish cattle breeds, namely Anatolian Black (AB), East Anatolian Red (EAR) and Zavot (ZAV), and to conduct phylogenetic relationship analyses to obtain deeper information on their genetic origin and breeding history by comparison of 6 taurine and 11 indicine breeds, together with 66 polymorphic sites, a total of 31 haplotypes, of which 15, 10 and 6 were detected in AB, EAR and ZAV, respectively. Mean nucleotide and haplotype diversity were 0.01 and 0.891, respectively, whereas the genetic differentiation derived from Wright's $F_{\mathrm{ST}}$ index was 0.174 across the breeds. A significant level of total variation (17.42 %) was observed among breeds in molecular variance analysis. Six main haplogroups (T, T1, T2, T3, Q and I2) were detected in Anatolian cattle populations, where T3 was the most frequent among breeds (43.55 %), whereas I2, an indicine specific haplogroup, was observed only in ZAV. At the breed level, phylogenetic analyses supported by 198 sequences of 17 cattle breeds and 3 outgroup species retrieved from the GenBank clustered native Turkish cattle breeds with the taurine group rather than the indicine one, as expected. However, indicine admixture at low frequency (8.89 %) was detected in the ZAV breed for the first time due to more likely gene flow from indicine cattle breeds raised in neighbour countries, particularly Iran. This finding should be further investigated in all native Turkish and indicine cattle breeds from nearby countries to clarify gene flow and indicine admixture in Anatolian cattle.

## 1 Introduction

In Türkiye, cattle farming is a part of daily life in rural areas to meet society's demand for milk and beef. Originated from *Bos taurus*, six native cattle breeds, namely Anatolian Black (AB), East Anatolian Red (EAR), Zavot (ZAV), East Anatolian Yellow (EAY), South Anatolian Red (SAR) and Turkish Grey Steppe (TGS), have been exclusively reared by smallholder farmers for centuries. However, particularly after World War II, local breeds could not meet all the demand for animal-derived products for the rapidly increasing human population. Therefore, the government initiated to import of globally widespread high-production (cosmopolitan) cattle breeds such as Holstein Friesian (HF), Brown Swiss (BS), Simmental (SIM), Jersey (JER), Hereford (HER) and Aberdeen Angus (ANG) from other countries between 1925 and 1970 (Koç, 2016). Compared to native Turkish cattle breeds, farmers have adopted cosmopolitan ones for their higher yield capacities (Demir et al., 2021). This preference has decreased effective population size and genetic diversity among native Turkish cattle breeds (Demir and Balcioğlu, 2019). Genetic diversity, on the other hand, plays a crucial role in the adaptation process against changing environmental conditions such as climate change, diseases, drought, forage and water scarcity. Indeed, today, priority is given to native livestock populations in conservation programmes across the world since local breeds may carry unique genotype combinations and genetic variants, which give opportunities to breeders to conduct selection practices against these environmental challenges (Karsli et al., 2022).

Thanks to developing genotyping methods and statistical approaches, genetic diversity could be easily estimated at different levels, such as from a single gene (Moravčíková et al., 2012) to genome-wide in cattle (Kasarda et al., 2020). Of these, mtDNA shows cytoplasmic inheritance, meaning that approximately 16 340 base pairs (bp) length of genetic information is passed to the next generations only by the maternal line. This unique type of inheritance has allowed scientists to clarify cattle evolution, domestication and migration processes (Troy et al., 2001). Moreover, the mtDNA D-loop region has been analysed to reveal genetic diversity in various cattle breeds since the mutation rate is higher compared to nuclear DNA (Kusumaningrum et al., 2020). Via domestication, the wild auroch (*Bos primigenius*) diverged into two different genetic strains such as *Bos taurus* (also known as taurine) and *Bos indicus* (also called indicine or zebu) (Utsunomiya et al., 2019). Although these strains differ in morphology, physiology, behaviour, and genetics, they could be easily distinguished by morphology in which *Bos indicus* is of hump phenotype, while *Bos taurus* is phenotypically humpless. mtDNA enables scientists to assess genetic diversity and estimate the admixture level of taurine and indicine in local cattle breeds since numerous sequence data deposited in the GenBank could be used for phylogenetic relationship analyses. In this regard, this study aimed to reveal genetic diversity, haplogroup classification and maternal admixture level in three native Turkish cattle breeds via mtDNA D-loop region.

## 2 Material and methods

### 2.1 Sample collection and DNA extraction

A total of 62 blood samples belonging to AB ( n=24 ), EAR ( n=23 ) and ZAV ( n=15 ) were collected from the jugular vein of animals into vacutainer tubes containing EDTA. AB was sampled from representative herds reared in Kütahya and Antalya provinces, while EAR and ZAV were sampled from Erzurum and Kars provinces, respectively. In the sampling strategy, pedigree records were utilised to choose unrelated animals for AB and EAR breeds to eliminate inbreeding. Unfortunately, no pedigree records were available for the ZAV breed, the lowest effective population among native Turkish cattle breeds and reared only in Kars province. Through oral interviews with farmers, only 15 unrelated animals were detected in order to eliminate inbreeding. Genomic DNA, including mtDNA extracted via the salting-out method described by Miller et al. (1988), was optimised at 50 ng mL ${}^{-1}$ concentration for PCR amplification.

### 2.2 Amplification and sequencing of mtDNA D-loop region

An 1138 bp fragment of mtDNA was amplified using primers CAP-F: 5 ${}^{\prime }$ -CCTAAGACTCAAGGAAGAAACTGC-3 ${}^{\prime }$ and CAP-R: 5 ${}^{\prime }$ -AACCTAGAGGGCATTCTCACTG- ${}^{\prime }$ 3 ${}^{\prime }$ (Achilli et al., 2008), corresponding to the positions 1518–517 in the sequence of the complete bovine mitochondrial genome (V00654). PCR was performed in 50 µ L reaction volume with 50 ng template DNA, 5 µ L 10X reaction buffer, 0.6 mM dNTPs, 2.5 mM MgCl ${}_{2}$ , 10 pM of each primer, 1 U of Taq DNA polymerase (GeNet Bio, South Korea) and 31.25 µ L nuclease-free water. PCR amplification was carried out in initial denaturation at 94 ${}^{\circ }$ C for 10 min, followed by 31 cycles at 94 ${}^{\circ }$ C for 40 s, at 63 ${}^{\circ }$ C for 40 s and 72 ${}^{\circ }$ C for 40 s. The final extension was applied at 72 ${}^{\circ }$ C for 10 min. Agarose gel electrophoresis was utilised to confirm PCR products which were further sequenced using the primer ${}^{\prime }$ 5 ${}^{\prime }$ -CCCCAAAGCTGAAGTTCTAT- ${}^{\prime }$ 3 ${}^{\prime }$ , as previously described (Achilli et al., 2008) by using a PCR purification kit (OMEGA Bio-tek Inc., Doraville, GA, USA) together with ABI 3100 Genetic Analyzer Sequencer. All sequences retrieved from the sequencer were optimised at 897 bp length by using CLC Sequence Viewer 8 program (https://www.qiagenbioinformatics.com, last access: 20 January 2023) and deposited into GenBank with accession numbers ON807611–ON807672.

### 2.3 Data preparation and statistical analyses

Most commonly preferred genetic diversity parameters such as nucleotide diversity ( π ), haplotype diversity ( $H_{d}$ ), number of haplotypes ( h ), number of polymorphic sites (NPS), number of monomorphic sites (NMS) and average number of nucleotide differences ( k ), as well as $F_{\mathrm{ST}}$ fixation index, were calculated by DnaSP v.6 (Rozas et al., 2017) with default settings. The MitoToolPy program (Peng et al., 2015) was run in a Python environment in order to detect haplogroups in each sample via pre-set options such as species (cattle) and region (dloop). Haplogroup classification of the detected haplotypes was visualised by median-joining network (MJ) analysis run in PopART 1.7 software (Leigh and Bryant, 2015) with default parameters. Analysis of molecular variance (AMOVA) was carried out to evaluate total genetic variation among and within populations via Arlequin 3.5.2.2 (Excoffier and Lischer, 2010) with 1000 permutations. The detailed information on the detected haplotypes and haplogroups in breed and sampling locations is summarised in File S1 in the Supplement.

A total of 125, 70 and 3 sequences belonging to cosmopolitan taurine (Aberdeen Angus, Brown Swiss, Hereford, Holstein Friesian, Jersey and Simmental), indicine (Bachaur, Gangatiri, Kenkatha, Kherigarh, Purnea, Shahabadi, Bhutanese, Myanmar, Iranian, Iraqi and Nellore) and outgroup species (bison, goat and sheep) were retrieved from GenBank database (Hiendleder, 1998; Steinborn et al., 2002; Hansen et al., 2003; Pietro et al., 2003; Slate and Phua, 2003; Lin et al., 2007; Achilli et al., 2008; Hiendleder et al., 2008; Ginja et al., 2010; Seroussi and Yakobson, 2010; Sharma et al., 2015; Lwin et al., 2018) and summarised in File S2. The geographic origins of taurine samples were Argentina (ANG and HER), Israel (ANG, HER, HF, SIM), Canada (BS, HF and JER), New Zealand (HF and JER) and USA (JER). In contrast, indicine samples originated from India (Bachaur, Gangatiri, Kenkatha, Kherigarh, Purnea and Shahabadi), Myanmar, Iran, and Iraq. These sequences were combined with our 62 sequences and optimised at 450 bp via the CLC Sequence Viewer 8 program (https://www.qiagenbioinformatics.com, last access: 20 January 2023) to explain the genetic origin and breeding history of native Turkish cattle breeds via phylogenetic relationship analyses. Neighbour-joining (NJ) and MJ analyses were utilised to visualise the distribution of the Anatolian cattle samples into taurine, indicine and outgroup clades. NJ tree analysis was conducted per individual and breed levels based on the Jukes–Cantor method with 1000 bootstrap replicates via MEGA 11 software (Kumar et al., 2008). MJ analysis was performed by PopART 1.7 software (Leigh and Bryant, 2015) with default parameters. DnaSP v.6 (Rozas et al., 2017) was used to perform haplotype classification to deepen knowledge of the genetic background of Anatolian cattle by comparison of each sequence with the haplotypes reported in 6 taurine and 11 indicine cattle breeds simultaneously. The level of admixture among taurine, indicine and Anatolian cattle breeds was estimated based on the observed proportion of shared haplotypes. The detailed information on the unique and shared sequences between Anatolian and other cattle (taurine and indicine) breeds is summarised in File S3.

## 3 Results

### 3.1 Genetic diversity and haplogroup classification

A total of 31 haplotypes (50 %) were detected with a mean of 0.010 and 0.891 nucleotide and haplotype diversity, respectively. The average NPS and NMS were 66 and 831, across all breeds. At the breed level, the highest genetic diversity parameters were observed in AB, except for NPS. Although the number of haplotypes was comparatively low, the highest NPS was detected in ZAV due to higher sequence variations (Table 1).

**Table 1:** An overview of genetic diversity parameters in studied breeds.

Breed	π	$H_{d}$	h	NPS	NMS	k
AB	0.005	0.935	15	24	837	4.612
EAR	0.004	0.881	10	18	879	3.984
ZAV	0.003	0.857	6	50	847	20.990
All population	0.010	0.891	31	66	831	9.423

π : nucleotide diversity; $H_{d}$ : haplotype diversity; h : number of haplotypes; NPS: number of polymorphic sites; NMS: number of monomorphic sites; k : average number of nucleotide differences.

All detected haplotypes turned out to be breed-specific (File S1), of which three haplotypes belonging to the AB breed (Hap_2, Hap_3, Hap_4) were common in two geographic locations known as Kütahya and Antalya. The highest and lowest number of haplotypes were detected in AB ( n=15 ) and ZAV ( n=6 ) breeds, respectively. The frequency of the haplotypes was generally low, in which Hap_24 was of the highest frequency (19.35 %), followed by Hap_18 with a frequency of 16.13 %. A total of 17 haplotypes (Hap_1, Hap_5, Hap_7–15, Hap_19, Hap_21–23, Hap_25 and Hap_31) were observed only in single samples with low frequencies (3.22 %).

Using a comparison algorithm of known haplogroups, the MitoToolPy program (Peng et al., 2015) assigned 31 haplotypes of native Turkish cattle into 6 main clades (T, T1, T2, T3, Q and I2) (Table 2) as well as several sub-clades such as T1b, T1b1, T1c, T2b, T3d, T3h, T3o, T3p and T3q (File S1). Of these haplogroups, T1, T3 and Q were detected in all breeds, whereas haplogroup T2 was present in both AB and EAR. Haplogroup T and I2 were detected only in AB and ZAV, respectively. T3 was the most common haplogroup (27 samples), while haplogroup T was detected in a sample from the AB breed.

**Table 2:** Haplogroup distribution of individuals of native Turkish cattle sequences.

Breed	T	T1	T2	T3	Q	I2
AB	1	6	7	9	1	–
EAR	–	4	6	12	1	–
ZAV	–	3	–	6	2	4
Total	1	13	13	27	4	4

**Figure 1:**
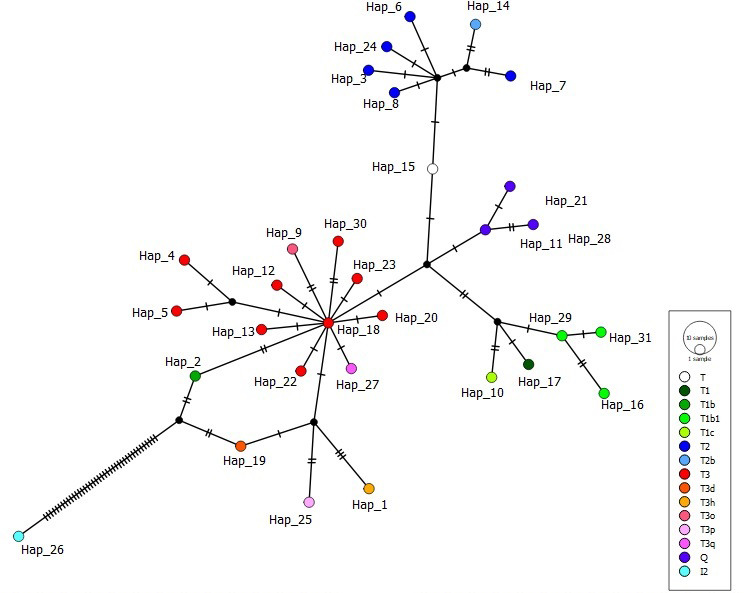
Median joining network of 31 haplotypes detected in three native Turkish cattle breeds based on 897 bp mtDNA D-loop region sequences (colours in circles indicate haplogroup assignment).

It is known that indicine cattle breeds possessing hump phenotype carry two main haplogroups known as I1 and I2, according to mtDNA D-loop information (Manomohan et al., 2021). Surprisingly, haplogroup I2 was detected in a haplotype (covering four samples in total) belonging to the ZAV breed, which is phenotypically humpless. In order to deepen knowledge of haplogroup I2, all haplotypes were screened via MJ network analysis in which the haplotype belonging to I2 differed from the nearest haplotypes (Hap_2 and Hap_19) by 40 mutation variations (Fig. 1).

$F_{\mathrm{ST}}$ fixation index ranged from 0.044 (AB-EAR) to 0.179 (EAR-ZAV), indicating that genetic differentiation between AB and EAR was low compared to ZAV (data not shown). However, significant genetic differentiation was observed between breeds by AMOVA analysis in which a large part of genetic variation was found within populations (82.58 %), while 17.42 % of the total variation was attributed to genetic differentiation among breeds (Table 3).

**Table 3:** AMOVA analysis in three native Turkish cattle breeds.

Source of variation	Degree of freedom	Sum of squares	Variance components	Percentage (%)
Among populations	2	43.602	0.871 Va	17.42
Within populations	59	243.801	4.132 Vb	82.58
Total	61	287.403		
Fixation index $F_{\mathrm{ST}}$	0.174			

### 3.2 Phylogenetic relationships analyses

**Figure 2:**
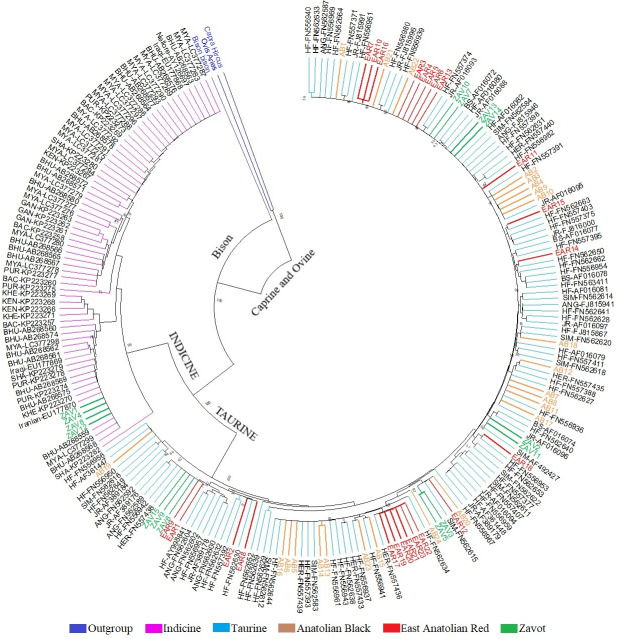
Neighbour joining analysis at the individual level via 450 bp mtDNA D-loop region sequences of NCBI database (taurine, indicine and outgroup species) and three native Turkish cattle breeds. The sequences retrieved from NCBI database were renamed based on GenBank number plus breed abbreviation as follows: Aberdeen Angus: ANG, Brown Swiss: BS; Hereford: HER; Holstein Friesian: HF; Jersey: JR; Simmental: SIM; Bachaur: BAC; Bhutanese: BHU; Gangatiri: GAN; Kenkatha: KEN; Kherigarh: KHE; Myanmar: MYA, Purnea: PUR and Shahabadi: SHA.

**Figure 3:**
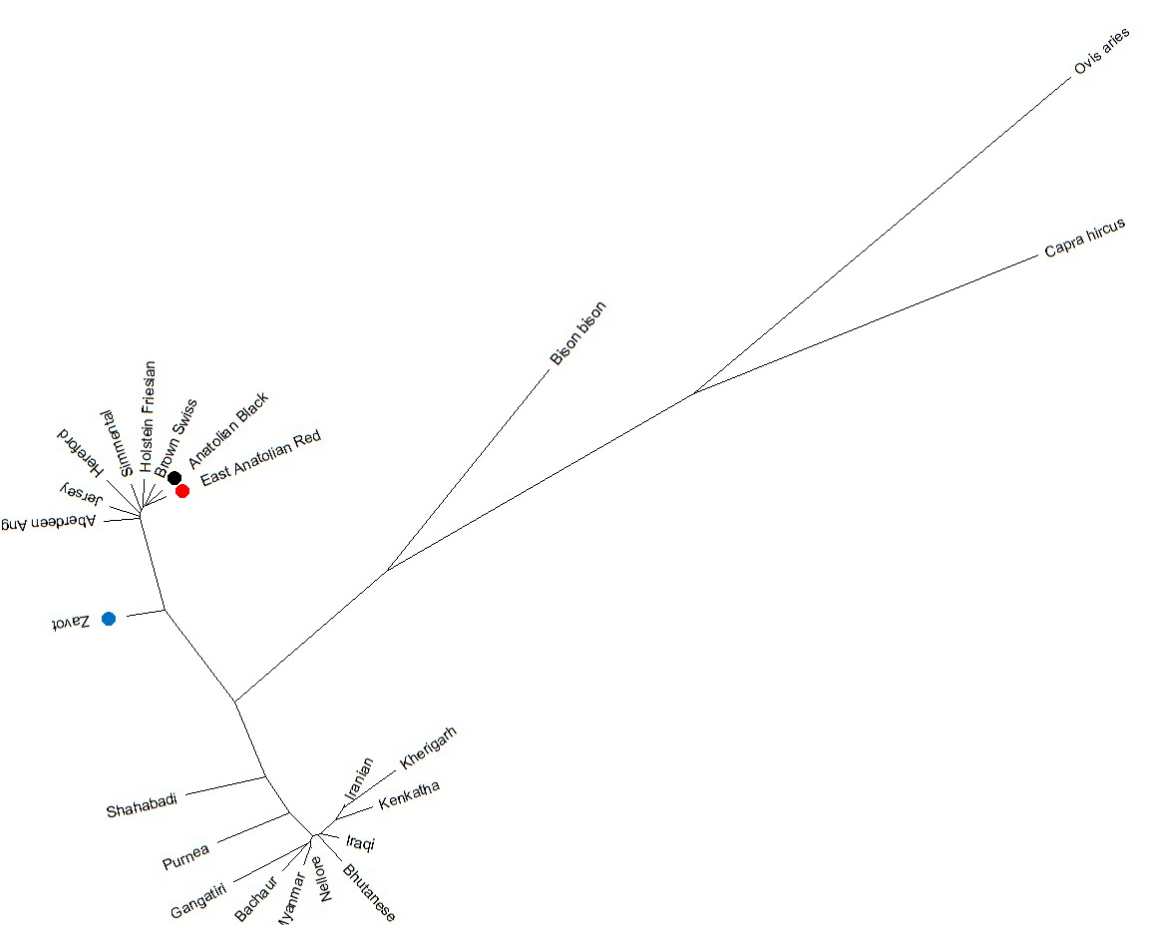
Neighbour joining analysis at breed level via 450 bp mtDNA D-loop region sequences of NCBI database (taurine, indicine and outgroup species) and three native Turkish cattle breeds.

Benefitting from 260 sequences (62 native Turkish cattle breeds, 125 taurine, 70 indicine and 3 outgroups) optimised at 450 bp length, the NJ tree clearly separated outgroup, taurine and indicine sequences at both individual (Fig. 2) and breed levels (Fig. 3). At the individual level, AB and EAR samples were clustered with taurine samples, particularly HF, BS, and SIM breeds (Fig. 2). However, four samples of ZAV (ZAV1, ZAV4, ZAV8 and ZAV12) were assigned to the indicine clade located between Iranian and Bhutanese samples (Fig. 2). Similarly, NJ tree analysis clustered AB and EAR together close to HF, BS and SIM at the breed level (Fig. 3). On the other hand, ZAV, which is still closer to taurine than indicine clade, was genetically different from native Turkish and cosmopolitan cattle breeds.

**Figure 4:**
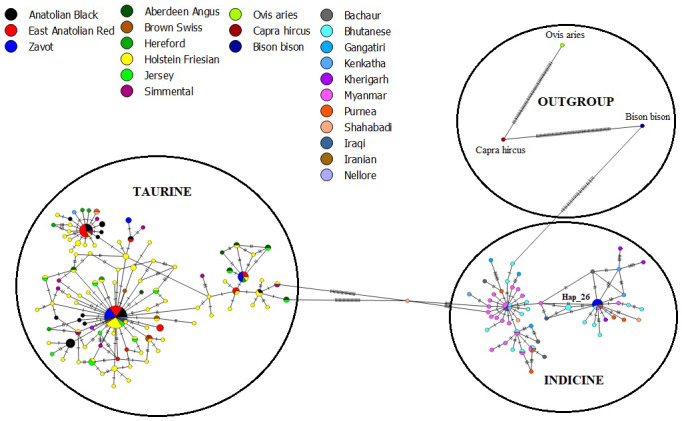
Median joining network of sequences from NCBI database (taurine, indicine and outgroup species) and three native Turkish cattle breeds via 450 bp mtDNA D-Loop region.

The results of NJ tree analyses were confirmed by MJ analysis, in which all samples of AB and EAR were connected with taurine haplotypes in at least one mutation event (Fig. 4). However, Hap_26, detected in four samples of ZAV, was assigned to the indicine group.

All sequences belonging to native Turkish cattle breeds were compared to cosmopolitan taurine and indicine breeds to detect the status of uniqueness and taurine and indicine admixture into Turkish cattle (File S3). A total of 15 (62.50 %), 6 (26.09 %) and 2 (13.33 %) sequences were specific to AB, EAR and ZAV breed, respectively (Table 4). No shared haplotypes were detected between AB and HER breed, while sequence similarity between native Turkish and cosmopolitan taurine breeds ranged from 9.56 % (HER) to 57.14 % (HF). Furthermore, no shared indicine sequences were detected in AB and EAR breeds, whereas four sequences of the ZAV breed were also present in three indicine breeds (Iranian, Myanmar, Bhutanese), indicating a low indicine admixture level (8.89 %) in ZAV (Table 4).

**Table 4:** Admixture level of taurine and indicine breeds in native Turkish cattle breeds.

Breed	n	Unique	Taurine ( n – %)						Indicine ( n – %)		
		( n – %)	ANG	BS	HER	HF	JER	SIM	Iranian	MYA	BHU
AB	24	15 – 62.50	5 – 20.83	5 – 20.83	–	9 – 37.5	5 – 20.83	8 – 33.33	–	–	–
EAR	23	6 – 26.09	6 – 26.09	7 – 30.43	2 – 8.70	17 – 73.91	6 – 26.09	12 – 52.17	–	–	–
ZAV	15	2 – 13.33	6 – 40.00	6 – 40.00	3 – 20.00	9 – 60.00	6 – 40.00	6 – 40.00	4 – 26.66	4 – 26.66	4 – 26.66
Mean	–	7.6 – 84.53	5.6 – 28.98	6 – 30.42	1.6 – 9.56	23.6 – 57.14	5.6 – 28.97	8.6 – 41.83	1.3 – 8.89	1.3 – 8.89	1.3 – 8.89

AB: Anatolian Black, EAR: East Anatolian Red, ZAV: Zavot, ANG: Aberdeen Angus, BS: Brown Swiss, HER: Hereford, HF: Holstein Friesian, JER: Jersey, SIM: Simmental, MYA: Myanmar, BHU: Bhutanese.

## 4 Discussion

### 4.1 Genetic diversity and haplogroup classification in native Turkish cattle breeds

Despite the low number of samples, a relatively high level of genetic diversity was detected in native Turkish cattle breeds in which AB was of the highest nucleotide and haplotype diversity. Indeed, AB is the most geographically distributed and preferred among native breeds, while ZAV is raised in the limited region of eastern Türkiye. As expected, nucleotide diversity was higher in native Turkish cattle breeds than in a previous study conducted on several cosmopolitan cattle breeds (ANG, BS, HF, JER and SIM) (Dorji et al., 2022). The long-term of artificial selection practices to increase phenotypic performance, such as milk yield, could lead to reduced genetic diversity and the extension of genomic homozygosity in pure cosmopolitan cattle breeds (Kelleher et al., 2017). Similarly, genetic diversity parameters were higher in Anatolian cattle compared to several Indonesian (Sragen Black) and Chinese (Qinchuan, Nanyang, Jianxian, Zaosheng, Menggu, Enshi, Xianan) cattle breeds (Yan et al., 2019; Kusumaningrum et al., 2020) due to geographic proximity of Türkiye to domestication centre. Naturally, genetic diversity is expected to decline as the distance from the domestication centres increases, mainly because a small percentage of the gene pool is distributed to other regions by human-mediated migration. High genetic diversity was also reported by Doğan et al. (2017), who revealed mtDNA diversity in all native Turkish cattle breeds ( n=279 ) via the D-loop region. Nucleotide diversity was reported to range from 0.013 (TGS) to 0.018 (EAR), as well as haplotype diversity varying between 0.983 (TGS) and 0.998 (SAY). Another study carried out by Özdemir and Dogru (2009) showed that haplotype diversity was 1.00 in four native Turkish cattle breeds with high nucleotide diversity ranging from 0.003 (TGS) to 0.006 (AB).

In this study, six main haplogroups were detected in native Turkish cattle breeds, in which T3 was the most distributed among both breeds and geographic locations. Troy et al. (2001) highlighted that T3 was the main haplogroup in European cattle. Results of higher genetic diversity, as well as haplogroup distribution and archaeological evidence, support the idea that taurine cattle were introduced to Europe from the domestication centre via different routes, among which Anatolia is both a part of the Fertile Crescent and has always been a trade route between Asia and Europe continents throughout the history. Therefore, it is more likely that taurine cattle were introduced to several countries by human-mediated migration from the Middle East to Europe via the Anatolia route.

### 4.2 Phylogenetic relationships and indicine admixture in native Turkish cattle breeds

All phylogenetic relationship analyses revealed three distinct clusters: taurine, indicine and outgroup, in which native Turkish cattle breeds were assigned to the taurine clade. This finding confirms the genetic origin of native Turkish cattle breeds which originated from *Bos taurus*. Phylogenetic relationships between native Turkish and other cattle breeds were also assessed by Özdemir and Dogru (2009) and Doğan et al. (2017). Native Turkish cattle breeds were reported to be clearly distinct from cattle breeds raised in India, Africa and Japan, while they were located close to European cattle breeds such as HF, SIM, BS and HER (Özdemir and Dogru, 2009). Indeed, in the present study, a high level of shared sequences was detected between native Turkish cattle and cosmopolitan breeds, particularly HF (57.14 %). It is not surprising because with the introduction of cosmopolitan cattle breeds to Türkiye in 1925, farmers, who adopted to raise high yielding breeds, initiated non-systematic crossbreeding practices between native and cosmopolitan breeds in order to increase milk and beef yield. This kind of rearing may lead to genetic admixture and gene flow between native Turkish and European-origin cattle breeds. Xuan et al. (2010) also preferred mtDNA D-loop variation to reveal the phylogenetic relationships in Romanian cattle breeds (Romanian Black Spotted, Romanian Brown and Romanian Grey Steppe) by comparison with several taurine and indicine samples from GenBank. As detected in the current study, the authors reported that Romanian cattle breeds clustered with taurine clade particularly close to HF, SIM and Grey cattle rather than indicine samples (Xuan et al., 2010).

Indicine admixture (10 %) was previously reported in EAR breed via mtDNA data by Edwards et al. (2007), while this paper is the first report to detect indicine admixture (8.89 %) in ZAV breed by mtDNA D-loop region. Hap_26, detected in four ZAV samples, was assigned to an indicine-specific haplogroup known as I2. Several controversial hypotheses are available about the genetic origin of ZAV in the literature. Historical records indicate that ZAV, which is a crossbreed, was introduced to Ardahan and Kars provinces by human migration from Tsarist Russia between 1850–1900 (Ortaylı, 1978; Arınç, 2018). Additionally, it is reported that ZAV was crossed with Ukrainian Steppe, Simmental, Brown Swiss and East Anatolian Red during both the same period and geographic locations (Arslan et al., 2015; Boğa Kuru and Kırmızıbayrak, 2020). Here, we describe an alternative explanation of the presence of I2 haplogroup and the genetic background of the ZAV breed.

Although taurine and indicine cattle were domesticated at different times and geographic locations, it is known that they were introduced to other countries by human migration, resulting in crossbreeding events between taurine and indicine cattle in several geographic locations. Iran and Iraq are home to several indicine cattle breeds such as Sistani, Mazandarani, Taleshi, Najdi and Jenoubi (Karimi et al., 2016; Alshawi et al., 2019). The presence of an indicine-specific haplogroup (I2) implies the possibility of crossbreeding events between ZAV and indicine cattle breeds in the past. Indeed, with the geographic distribution of the ZAV breed, previous studies on cattle evolution and migration routes (Utsunomiya et al., 2019) enable us to conclude that the crossbreeding possibility of ZAV and indicine Iranian cattle populations seems logical. This possibility may allow the ZAV breed to become genetically different from the other native Turkish cattle breeds and possess haplogroup I2.

## 5 Conclusions

In this study, genetic variations and haplogroup distribution were assessed in three Anatolian cattle breeds via mtDNA D-loop region in which a high level of genetic diversity was observed. This finding supports the idea that cattle populations geographically close to the domestication centre not only conserve more ancestral genetic variations but also they are a gene pool for the other cattle breeds raised across the world. Conservation of high genetic diversity will be helpful for animals to face environmental challenges and allow farmers to carry out different selection practices in the future. Besides, phylogenetic relationship analyses revealed that Anatolian cattle shared genetic similarities with cosmopolitan taurine breeds at the mtDNA level due to originating from the same ancestor and crossbreeding practices. However, indicine-specific haplogroup I2 was detected in ZAV for the first time, which should be further analysed together with Iranian and Iraqi cattle breeds to clarify gene flow and migration. Besides, the farmers keep no pedigree records for ZAV breed, which prevents the scientists from estimating the effective population size and detecting unrelated animals for molecular studies. Moreover, pedigree records could be updated by farmers in order to achieve breeding and selection practices as well as to prevent genetic erosion in the ZAV breed.

Unfortunately, mtDNA analyses in native Turkish cattle breeds are still scarce. Moreover, present studies are limited by mtDNA D-loop region corresponding to a small part of mtDNA. Here, we highly recommend further studies focusing on entire mitogenome diversity with a higher sample size to clarify not only genetic diversity in cattle breeds but also to enlighten cattle domestication and migration process.

## Supplement

10.5194/aab-66-31-2023-supplement The supplement related to this article is available online at: https://doi.org/10.5194/aab-66-31-2023-supplement.

## Data Availability

mtDNA sequences used in this study were deposited into GenBank (, last access: 20 January 2023) with accession number ON807611 ()–ON807672 () (Demir et al., 2023).
