# Supplementary material for: Mitochondrial DNA diversity of D-loop region in three native Turkish cattle breeds
Source: Arch Anim Breed. 2023 Jan 24;66(1):31–40. doi: 10.5194/aab-66-31-2023 (PMC9901521; doi:10.5194/aab-66-31-2023)
Supplement: The supplement related to this article is available online at: https://doi.org/10.5194/aab-66-31-2023-supplement. [file aab-66-31-supplement.zip › File S3.pdf]

Supplementary file 3. Unique and shared-sequences between native Turkish and cosmopolitan-taurine and indicine cattle

Samples	Unique	Aberdeen Angus	Brown Swiss	Hereford	Holstein Friesian	Jersey	Simmental	Iranian	Myanmar	Bhutanese
AB1	Yes	-	-	-	-	-	-	-	-	-
AB2	Yes	-	-	-	-	-	-	-	-	-
AB3	Yes	-	-	-	-	-	-	-	-	-
AB4	Yes	-	-	-	-	-	-	-	-	-
AB5	No	-	-	-	FN562644	-	FN562612	-	-	-
AB6	No	-	-	-	FN562644	-	FN562612	-	-	-
AB7	No	FJ815946	AF016072	-	AF016082	AF016088	AF492427	-	-	-
AB8	No	FJ815946	AF016072	-	AF016082	AF016088	AF492427	-	-	-
AB9	Yes	-	-	-	-	-	-	-	-	-
AB10	Yes	-	-	-	-	-	-	-	-	-
AB11	No	FJ815946	AF016072	-	AF016082	AF016088	AF492427	-	-	-
AB12	Yes	-	-	-	-	-	-	-	-	-
AB13	Yes	-	-	-	-	-	-	-	-	-
AB14	Yes	-	-	-	-	-	-	-	-	-
AB15	Yes	-	-	-	-	-	-	-	-	-
AB16	No	-	-	-	FN562644	-	FN562612	-	-	-
AB17	No	FJ815946	AF016072	-	AF016082	AF016088	AF492427	-	-	-
AB18	Yes									
AB19	No	-	-	-	AF361447	-	-	-	-	-
AB20	Yes	-	-	-	-	-	-	-	-	-
AB21	Yes	-	-	-	-	-	-	-	-	-
AB22	No	FJ815946	AF016072	-	AF016082	AF016088	AF492427	-	-	-
AB23	Yes	-	-	-	-	-	-	-	-	-
AB24	Yes	-	-	-	-	-	-	-	-	-
EAR1	No	-	-	FN557438	FN556942	-	-	-	-	-
EAR2	No	-	-	-	FN562630	-	-	-	-	-
EAR3	No	FJ815946	AF016072	-	AF016082	AF016088	AF492427	-	-	-
EAR4	No	FJ815946	AF016072	-	AF016082	AF016088	AF492427	-	-	-

EAR5	No	FJ815946	AF016072	-	AF016082	AF016088	AF492427	-	-	-
EAR6	No	FJ815946	AF016072	-	AF016082	AF016088	AF492427	-	-	-
EAR7	Yes	-	-	-	-	-	-	-	-	-
EAR8	No	-	-	-	FN562630	-	-	-	-	-
EAR9	No	-	-	FN557438	FN556942	-	-	-	-	-
EAR10	Yes	-	-	-	-	-	-	-	-	-
EAR11	No	FJ815946	AF016072	-	AF016082	AF016088	AF492427	-	-	-
EAR12	Yes	-	-	-	-	-	-	-	-	-
EAR13	No	FJ815946	AF016072	-	AF016082	AF016088	AF492427	-	-	-
EAR14	No	-	AF016078	-	FN563411	-	-	-	-	-
EAR15	Yes	-	-	-	-	-	-	-	-	-
EAR16	Yes	-	-	-	-	-	-	-	-	-
EAR17	No	-	-	-	FN562644	-	FN562612	-	-	-
EAR18	Yes	-	-	-	-	-	-	-	-	-
EAR19	No	-	-	-	FN562644	-	FN562612	-	-	-
EAR20	No	-	-	-	FN562644	-	FN562612	-	-	-
EAR21	No	-	-	-	FN562644	-	FN562612	-	-	-
EAR22	No	-	-	-	FN562644	-	FN562612	-	-	-
EAR23	No	-	-	-	FN562644	-	FN562612	-	-	-
ZAV1	No	-	-	-	-	-	-	EU177870	LC377299	AB268559
ZAV2	No	FJ815946	AF016072	-	AF016082	AF016088	AF492427	-	-	-
ZAV3	Yes	-	-	-	-	-	-	-	-	-
ZAV4	No	-	-	-	-	-	-	EU177870	LC377299	AB268559
ZAV5	Yes	-	-	-	-	-	-	-	-	-
ZAV6	No	-	-	FN557438	FN556942	-	-	-	-	-
ZAV7	No	FJ815946	AF016072	-	AF016082	AF016088	AF492427	-	-	-
ZAV8	No	-	-	-	-	-	-	EU177870	LC377299	AB268559
ZAV9	No	-	-	FN557438	FN556942	-	-	-	-	-
ZAV10	No	FJ815946	AF016072	-	AF016082	AF016088	AF492427	-	-	-
ZAV11	No	FJ815946	AF016072	-	AF016082	AF016088	AF492427	-	-	-
ZAV12	No	-	-	-	-	-	-	EU177870	LC377299	AB268559

ZAV13	No	FJ815946	AF016072	-	AF016082	AF016088	AF492427	-	-	-
ZAV14	No	FJ815946	AF016072	-	AF016082	AF016088	AF492427	-	-	-
ZAV15	No	-	-	FN557438	FN556942	-	-	-	-	-
