# Supplementary material for: Mitochondrial DNA diversity of D-loop region in three native Turkish cattle breeds
Source: Arch Anim Breed. 2023 Jan 24;66(1):31–40. doi: 10.5194/aab-66-31-2023 (PMC9901521; doi:10.5194/aab-66-31-2023)
Supplement: The supplement related to this article is available online at: https://doi.org/10.5194/aab-66-31-2023-supplement. [file aab-66-31-supplement.zip › File S1.pdf]

Supplementary file 1. Haplotype and haplogroup distribution based on breeds and geographic locations

**Haplotypes**	**Haplogroup**	**Breeds**			**Sampling Locations**			
		**AB (n)**	**EAR (n)**	**ZAV (n)**	**Kütahya**	**Antalya**	**Erzurum**	**Kars**
Hap_1	T3h	1	0	0	+	-	-	-
Hap_2	T1b	4	0	0	+	+	-	-
Hap_3	T2	2	0	0	+	+	-	-
Hap_4	T3	4	0	0	+	+	-	-
Hap_5	T3	1	0	0	-	+	-	-
Hap_6	T2	2	0	0	-	+	-	-
Hap_7	T2	1	0	0	-	+	-	-
Hap_8	T2	1	0	0	-	+	-	-
Hap_9	T3o	1	0	0	-	+	-	-
Hap_10	T1c	1	0	0	-	+	-	-
Hap_11	Q	1	0	0	-	+	-	-
Hap_12	T3	1	0	0	-	+	-	-
Hap_13	T3	1	0	0	-	+	-	-
Hap_14	T2b	1	0	0	-	+	-	-
Hap_15	T	1	0	0	-	+	-	-
Hap_16	T1b1	0	2	0	-	-	+	-
Hap_17	T1	0	2	0	-	-	+	-
Hap_18	T3	0	5	0	-	-	+	-
Hap_19	T3d	0	1	0	-	-	+	-
Hap_20	T3	0	3	0	-	-	+	-
Hap_21	Q	0	1	0	-	-	+	-
Hap_22	T3	0	1	0	-	-	+	-
Hap_23	T3	0	1	0	-	-	+	-
Hap_24	T2	0	6	0	-	-	+	-
Hap_25	T3p	0	1	0	-	-	+	-
Hap_26	**I2**	0	0	4	-	-	-	+
Hap_27	T3q	0	0	2	-	-	-	+
Hap_28	Q	0	0	2	-	-	-	+
Hap_29	T1b1	0	0	2	-	-	-	+
Hap_30	T3	0	0	4	-	-	-	+
Hap_31	T1b1	0	0	1	-	-	-	+
**Total**	**-**	**15**	**10**	**6**	**4**	**14**	**10**	**6**
