# Supplementary material for: Mitochondrial DNA diversity of D-loop region in three native Turkish cattle breeds
Source: Arch Anim Breed. 2023 Jan 24;66(1):31–40. doi: 10.5194/aab-66-31-2023 (PMC9901521; doi:10.5194/aab-66-31-2023)
Supplement: The supplement related to this article is available online at: https://doi.org/10.5194/aab-66-31-2023-supplement. [file aab-66-31-supplement.zip › File S2.pdf]

Supplementary file 2. An overview of the sequences retrieved from GenBank database

**Breed**	**Accession Numbers**	**End Position (~bp)**	**Start Position (~bp)**	**Length (bp)**	**References**
Brown Swiss	AF016072	457	15695	798	Hansen et al., 2003
Brown Swiss	AF016073	457	15695	799	Hansen et al., 2003
Brown Swiss	AF016074	457	15695	800	Hansen et al., 2003
Brown Swiss	AF016075	457	15695	798	Hansen et al., 2003
Brown Swiss	AF016076	457	15695	799	Hansen et al., 2003
Brown Swiss	AF016077	457	15695	800	Hansen et al., 2003
Brown Swiss	AF016078	457	15695	797	Hansen et al., 2003
Holstein Friesian	AF016079	457	15695	798	Hansen et al., 2003
Holstein Friesian	AF016080	457	15695	801	Hansen et al., 2003
Holstein Friesian	AF016081	457	15695	798	Hansen et al., 2003
Holstein Friesian	AF016082	457	15695	799	Hansen et al., 2003
Holstein Friesian	AF016083	457	15695	798	Hansen et al., 2003
Holstein Friesian	AF016084	457	15695	799	Hansen et al., 2003
Holstein Friesian	AF016085	457	15695	799	Hansen et al., 2003
Holstein Friesian	AF016086	457	15695	798	Hansen et al., 2003
Holstein Friesian	AF016087	457	15695	799	Hansen et al., 2003
Jersey	AF016088	457	15695	799	Hansen et al., 2003
Jersey	AF016089	457	15695	796	Hansen et al., 2003
Jersey	AF016090	457	15695	799	Hansen et al., 2003
Jersey	AF016091	457	15695	800	Hansen et al., 2003
Jersey	AF016092	457	15695	802	Hansen et al., 2003
Jersey	AF016093	457	15695	800	Hansen et al., 2003
Jersey	AF016094	457	15695	800	Hansen et al., 2003
Jersey	AF016095	457	15695	800	Hansen et al., 2003
Jersey	AF016096	457	15695	799	Hansen et al., 2003

Jersey	AF016097	457	15695	800	Hansen et al., 2003
Aberdeen Angus	FJ815941	516	15737	910	Ginja et al., 2010
Holstein Friesian	FN562641	355	15921	733	Seroussi and Yakobson, 2010
Holstein Friesian	FN556952	355	15921	733	Seroussi and Yakobson, 2010
Simmental	FN562585				
Holstein Friesian	FJ815867	516	15737	910	Ginja et al., 2010
Simmental	FN562620	355	15921	733	Seroussi and Yakobson, 2010
Holstein Friesian	FN557398	355	15921	733	Seroussi and Yakobson, 2010
Simmental	FN562612			732	
Holstein Friesian	FN562661	355	15921	732	Seroussi and Yakobson, 2010
Holstein Friesian	AF361448	355	15921	918	Steinborn et al., 2002
Jersey	AF389179			948	Slate and Phua, 2003
Aberdeen Angus	FN562609	355	15921	733	Seroussi and Yakobson, 2010
Holstein Friesian	FN556942			732	
Aberdeen Angus	FJ815946	516	15737	909	Ginja et al., 2010
Holstein Friesian	FN562651	355	15921	733	Seroussi and Yakobson, 2010
Holstein Friesian	FN556936	355	15921	733	Seroussi and Yakobson, 2010
Holstein Friesian	FN556981	355	15921	733	Seroussi and Yakobson, 2010
Simmental	FN562584	355	15921	733	Seroussi and Yakobson, 2010
Aberdeen Angus	FN562600	355	15921	733	Seroussi and Yakobson, 2010
Jersey	AF389178				
Holstein Friesian	FN557371	355	15921	733	Seroussi and Yakobson, 2010
Jersey	FJ815991	516	15737	909	Ginja et al., 2010
Holstein Friesian	FN562649	355	15921	733	Seroussi and Yakobson, 2010
Simmental	FN562616				
Holstein Friesian	FN562644	355	15921	733	Seroussi and Yakobson, 2010
Holstein Friesian	AF361447	355	15921	918	Steinborn et al., 2002
Holstein Friesian	FN556951	355	15921	733	Seroussi and Yakobson, 2010
Holstein Friesian	FN556958	355	15921	733	Seroussi and Yakobson, 2010

Holstein Friesian	FN556969	355	15921	733	Seroussi and Yakobson, 2010
Aberdeen Angus	FN562602	355	15921	733	Seroussi and Yakobson, 2010
Holstein Friesian	FN562662	355	15921	732	Seroussi and Yakobson, 2010
Holstein Friesian	FN556966	355	15921	735	Seroussi and Yakobson, 2010
Aberdeen Angus	FN562589	355	15921	733	Seroussi and Yakobson, 2010
Jersey	AF389176	363	15792	979	Slate and Phua, 2003
Holstein Friesian	FN556980	355	15921	733	Seroussi and Yakobson, 2010
Jersey	FJ815996	516	15737	909	Ginja et al., 2010
Holstein Friesian	FN557380	355	15921	733	Seroussi and Yakobson, 2010
Holstein Friesian	FN556975	355	15921	733	Seroussi and Yakobson, 2010
Aberdeen Angus	FN562592	355	15921	733	Seroussi and Yakobson, 2010
Jersey	AF389184	363	15792	969	Slate and Phua, 2003
Simmental	AF492427	355	15921	910	Hiendleder et al., 2008
Holstein Friesian	FN556940	355	15921	733	Seroussi and Yakobson, 2010
Holstein Friesian	FN557387	355	15921	733	Seroussi and Yakobson, 2010
Holstein Friesian	FN557375	355	15921	732	Seroussi and Yakobson, 2010
Jersey	FJ816000	516	15737	908	Ginja et al., 2010
Aberdeen Angus	FN562604	355	15921	732	Seroussi and Yakobson, 2010
Holstein Friesian	AY998841	363	15792	911	Direct submission (Shanghai Institute of Medical Genetics)
Holstein Friesian	FN556974	355	15921	732	Seroussi and Yakobson, 2010
Holstein Friesian	FN556968	355	15921	733	Seroussi and Yakobson, 2010
Holstein Friesian	FN556959	355	15921	735	Seroussi and Yakobson, 2010
Simmental	FN562611	355	15921	734	Seroussi and Yakobson, 2010
Holstein Friesian	FN562646	355	15921	733	Seroussi and Yakobson, 2010
Hereford	FN557438	355	15921	733	Seroussi and Yakobson, 2010
Holstein Friesian	FN557404				
Simmental	FN562586	355	15921	733	Seroussi and Yakobson, 2010
Simmental	FN562618	355	15921	733	Seroussi and Yakobson, 2010

Holstein Friesian	FN557405	355	15921	732	Seroussi and Yakobson, 2010
Holstein Friesian	FN557393	355	15921	732	Seroussi and Yakobson, 2010
Holstein Friesian	FN556956	355	15921	732	Seroussi and Yakobson, 2010
Holstein Friesian	FN556937	355	15921	732	Seroussi and Yakobson, 2010
Holstein Friesian	FN556961	355	15921	732	Seroussi and Yakobson, 2010
Holstein Friesian	FN562634	355	15921	732	Seroussi and Yakobson, 2010
Hereford	FN557433	355	15921	732	Seroussi and Yakobson, 2010
Holstein Friesian	FN562663	355	15921	735	Seroussi and Yakobson, 2010
Holstein Friesian	FN557397	355	15921	734	Seroussi and Yakobson, 2010
Hereford	FN557440	355	15921	734	Seroussi and Yakobson, 2010
Holstein Friesian	FN557406	355	15921	733	Seroussi and Yakobson, 2010
Holstein Friesian	FN562638	355	15921	733	Seroussi and Yakobson, 2010
Holstein Friesian	FN556953	355	15921	733	Seroussi and Yakobson, 2010
Holstein Friesian	FN556954	355	15921	733	Seroussi and Yakobson, 2010
Holstein Friesian	FN562650	355	15921	733	Seroussi and Yakobson, 2010
Holstein Friesian	FN562639	355	15921	733	Seroussi and Yakobson, 2010
Holstein Friesian	FN562640	355	15921	733	Seroussi and Yakobson, 2010
Holstein Friesian	FN557408	355	15921	733	Seroussi and Yakobson, 2010
Holstein Friesian	FN562642	355	15921	733	Seroussi and Yakobson, 2010
Holstein Friesian	FN557413	355	15921	733	Seroussi and Yakobson, 2010
Simmental	FN562583	355	15921	733	Seroussi and Yakobson, 2010
Holstein Friesian	FN562627	355	15921	733	Seroussi and Yakobson, 2010
Holstein Friesian	FN562628	355	15921	733	Seroussi and Yakobson, 2010
Holstein Friesian	FN562629	355	15921	733	Seroussi and Yakobson, 2010
Holstein Friesian	FN562630	355	15921	733	Seroussi and Yakobson, 2010
Holstein Friesian	FN562633	355	15921	733	Seroussi and Yakobson, 2010
Holstein Friesian	FN556962	355	15921	733	Seroussi and Yakobson, 2010
Holstein Friesian	FN562652	355	15921	733	Seroussi and Yakobson, 2010
Holstein Friesian	FN557403	355	15921	733	Seroussi and Yakobson, 2010

Holstein Friesian	FN562653	355	15921	733	Seroussi and Yakobson, 2010
Holstein Friesian	FN556941	355	15921	733	Seroussi and Yakobson, 2010
Holstein Friesian	FN557414	355	15921	733	Seroussi and Yakobson, 2010
Holstein Friesian	FN556965	355	15921	733	Seroussi and Yakobson, 2010
Holstein Friesian	FN556943	355	15921	733	Seroussi and Yakobson, 2010
Holstein Friesian	FN556938	355	15921	733	Seroussi and Yakobson, 2010
Holstein Friesian	FN557401	355	15921	733	Seroussi and Yakobson, 2010
Aberdeen Angus	FN562587	355	15921	733	Seroussi and Yakobson, 2010
Holstein Friesian	FN556972	355	15921	733	Seroussi and Yakobson, 2010
Holstein Friesian	FN557373	355	15921	733	Seroussi and Yakobson, 2010
Holstein Friesian	FN557377	355	15921	733	Seroussi and Yakobson, 2010
Simmental	FN562614	355	15921	733	Seroussi and Yakobson, 2010
Simmental	FN562615	355	15921	733	Seroussi and Yakobson, 2010
Simmental	FN562622	355	15921	733	Seroussi and Yakobson, 2010
Holstein Friesian	FN556949	355	15921	733	Seroussi and Yakobson, 2010
Hereford	FN557435	355	15921	733	Seroussi and Yakobson, 2010
Holstein Friesian	FN562664	355	15921	733	Seroussi and Yakobson, 2010
Holstein Friesian	FN556950	355	15921	733	Seroussi and Yakobson, 2010
Holstein Friesian	FN557388	355	15921	733	Seroussi and Yakobson, 2010
Holstein Friesian	FN556982	355	15921	733	Seroussi and Yakobson, 2010
Holstein Friesian	FN556978	355	15921	733	Seroussi and Yakobson, 2010
Holstein Friesian	FN557391	355	15921	733	Seroussi and Yakobson, 2010
Holstein Friesian	FN557392	355	15921	732	Seroussi and Yakobson, 2010
Holstein Friesian	FN562635	355	15921	732	Seroussi and Yakobson, 2010
Holstein Friesian	FN556955	355	15921	732	Seroussi and Yakobson, 2010
Holstein Friesian	FN557395	355	15921	732	Seroussi and Yakobson, 2010
Holstein Friesian	FN556957	355	15921	732	Seroussi and Yakobson, 2010
Holstein Friesian	FN562648	355	15921	732	Seroussi and Yakobson, 2010
Holstein Friesian	FN557407	355	15921	732	Seroussi and Yakobson, 2010

Hereford	FN557439	355	15921	732	Seroussi and Yakobson, 2010
Holstein Friesian	FN556939	355	15921	732	Seroussi and Yakobson, 2010
Holstein Friesian	FN557411	355	15921	732	Seroussi and Yakobson, 2010
Holstein Friesian	FN562643	355	15921	732	Seroussi and Yakobson, 2010
Holstein Friesian	FN562632	355	15921	732	Seroussi and Yakobson, 2010
Holstein Friesian	FN562654	355	15921	732	Seroussi and Yakobson, 2010
Holstein Friesian	FN562655	355	15921	732	Seroussi and Yakobson, 2010
Holstein Friesian	FN562656	355	15921	732	Seroussi and Yakobson, 2010
Holstein Friesian	FN556967	355	15921	732	Seroussi and Yakobson, 2010
Holstein Friesian	FN557374	355	15921	732	Seroussi and Yakobson, 2010
Holstein Friesian	FN563411	355	15921	732	Seroussi and Yakobson, 2010
Holstein Friesian	FN556948	355	15921	732	Seroussi and Yakobson, 2010
Holstein Friesian	FN557385	355	15921	732	Seroussi and Yakobson, 2010
Hereford	FN557436	355	15921	732	Seroussi and Yakobson, 2010
Holstein Friesian	FN562631	355	15921	729	Seroussi and Yakobson, 2010
Bachaur	KP223257	56	15718	679	Sharma et al., 2015
Bachaur	KP223258	56	15718	679	Sharma et al., 2015
Bachaur	KP223259	56	15759	638	Sharma et al., 2015
Bachaur	KP223260	92	15777	656	Sharma et al., 2015
Gangatiri	KP223261	83	15764	660	Sharma et al., 2015
Gangatiri	KP223262	73	15718	696	Sharma et al., 2015
Gangatiri	KP223263	73	15795	619	Sharma et al., 2015
Gangatiri	KP223264	73	15756	658	Sharma et al., 2015
Kenkatha	KP223265	56	15711	686	Sharma et al., 2015
Kenkatha	KP223266	56	15715	682	Sharma et al., 2015
Kenkatha	KP223267	58	15719	680	Sharma et al., 2015
Kenkatha	KP223268	56	15711	686	Sharma et al., 2015
Kherigarh	KP223269	59	15741	659	Sharma et al., 2015
Kherigarh	KP223270	56	15718	679	Sharma et al., 2015

Kherigarh	KP223271	57	15718	680	Sharma et al., 2015
Kherigarh	KP223272	56	15718	679	Sharma et al., 2015
Purnea	KP223273	145	15713	773	Sharma et al., 2015
Purnea	KP223274	147	15719	769	Sharma et al., 2015
Purnea	KP223275	147	15719	769	Sharma et al., 2015
Purnea	KP223276	143	15632	852	Sharma et al., 2015
Purnea	KP223277	144	15629	856	Sharma et al., 2015
Purnea	KP223278	144	15632	853	Sharma et al., 2015
Shahabadi	KP223279	86	15718	709	Sharma et al., 2015
Shahabadi	KP223280	86	15718	709	Sharma et al., 2015
Shahabadi	KP223281	86	15718	709	Sharma et al., 2015
Shahabadi	KP223282	86	15718	709	Sharma et al., 2015
Iraqi	EU177868	363	15793	910	Achilli et al., 2008
Iraqi	EU177869	363	15793	910	Achilli et al., 2008
Iranian	EU177870	363	15793	910	Achilli et al., 2008
Nellore	AY126697	366	15795	911	Direct submission (Depto. de Tecnologia, FCAV, Universidade Estadual Paulista)
Myanmar cattle	LC377275	366	15795	911	Lwin et al., 2018
Myanmar cattle	LC377276	366	15795	911	Lwin et al., 2018
Myanmar cattle	LC377277	366	15795	911	Lwin et al., 2018
Myanmar cattle	LC377278	366	15795	911	Lwin et al., 2018
Myanmar cattle	LC377279	366	15795	911	Lwin et al., 2018
Myanmar cattle	LC377280	366	15795	911	Lwin et al., 2018
Myanmar cattle	LC377281	366	15795	911	Lwin et al., 2018
Myanmar cattle	LC377282	366	15795	911	Lwin et al., 2018
Myanmar cattle	LC377283	366	15795	912	Lwin et al., 2018
Myanmar cattle	LC377284	366	15795	911	Lwin et al., 2018
Myanmar cattle	LC377285	366	15795	911	Lwin et al., 2018
Myanmar cattle	LC377286	366	15795	911	Lwin et al., 2018

Myanmar cattle	LC377287	366	15795	911	Lwin et al., 2018
Myanmar cattle	LC377288	366	15795	911	Lwin et al., 2018
Myanmar cattle	LC377289	366	15795	910	Lwin et al., 2018
Myanmar cattle	LC377290	366	15795	910	Lwin et al., 2018
Myanmar cattle	LC377291	366	15795	910	Lwin et al., 2018
Myanmar cattle	LC377292	366	15795	910	Lwin et al., 2018
Myanmar cattle	LC377293	366	15795	910	Lwin et al., 2018
Myanmar cattle	LC377294	366	15795	910	Lwin et al., 2018
Myanmar cattle	LC377295	366	15795	910	Lwin et al., 2018
Myanmar cattle	LC377296	366	15795	910	Lwin et al., 2018
Myanmar cattle	LC377297	366	15795	911	Lwin et al., 2018
Myanmar cattle	LC377298	366	15795	911	Lwin et al., 2018
Myanmar cattle	LC377299	366	15795	911	Lwin et al., 2018
Myanmar cattle	LC377300	366	15795	910	Lwin et al., 2018
Myanmar cattle	LC377301	366	15795	911	Lwin et al., 2018
Bhutanese cattle	AB268559	366	15795	910	Lin et al., 2007
Bhutanese cattle	AB268560	366	15795	910	Lin et al., 2007
Bhutanese cattle	AB268561	366	15795	909	Lin et al., 2007
Bhutanese cattle	AB268562	366	15795	909	Lin et al., 2007
Bhutanese cattle	AB268563	366	15795	910	Lin et al., 2007
Bhutanese cattle	AB268564	366	15795	910	Lin et al., 2007
Bhutanese cattle	AB268565	366	15795	910	Lin et al., 2007
Bhutanese cattle	AB268566	366	15795	910	Lin et al., 2007
Bhutanese cattle	AB268567	366	15795	910	Lin et al., 2007
Bhutanese cattle	AB268568	366	15795	910	Lin et al., 2007
Bhutanese cattle	AB268569	366	15795	910	Lin et al., 2007
Bhutanese cattle	AB268570	366	15795	909	Lin et al., 2007
Bhutanese cattle	AB268571	366	15795	910	Lin et al., 2007
Bhutanese cattle	AB268572	366	15795	910	Lin et al., 2007

Bhutanese cattle	AB268573	366	15795	910	Lin et al., 2007
Bhutanese cattle	AB268574	366	15795	910	Lin et al., 2007
Bhutanese cattle	AB268575	366	15795	910	Lin et al., 2007
Bhutanese cattle	AB268576	366	15795	910	Lin et al., 2007
Bhutanese cattle	AB268577	366	15795	910	Lin et al., 2007
Bhutanese cattle	AB268578	366	15795	910	Lin et al., 2007
Bhutanese cattle	AB268579	366	15795	910	Lin et al., 2007
Bhutanese cattle	AB268580	366	15795	910	Lin et al., 2007
Bhutanese cattle	AB268581	366	15795	910	Lin et al., 2007
Bison bison	NC_012346	16319	1	16319	Achilli et al., 2008
Capra hircus	AF533441	16640	1	16640	Pietro et al., 2003
Ovis aries	NC_001941	16616	1	16616	Hiendleder, 1998
