# Supplementary material for: Mitochondrial DNA diversity of D-loop region in three native Turkish cattle breeds
Source: Arch Anim Breed. 2023 Jan 24;66(1):31–40. doi: 10.5194/aab-66-31-2023 (PMC9901521; doi:10.5194/aab-66-31-2023)
Supplement: The supplement related to this article is available online at: https://doi.org/10.5194/aab-66-31-2023-supplement. [file aab-66-31-supplement.zip › aab-66-31-2023-supplement-title-page.pdf]

Supplement of Arch. Anim. Breed., 66, 31–40, 2023
https://doi.org/10.5194/aab-66-31-2023-supplement
© Author(s) 2023. CC BY 4.0 License.

Open Access
Archives Animal Breeding

Supplement of

Mitochondrial DNA diversity of D-loop region in three native Turkish cattle breeds

Eymen Demir et al.

Correspondence to: Taki Karsli (takikarsli@ogu.edu.tr)

- aab-66-31-2023-supplement-title-page.pdf
- File S1.pdf
- File S2.pdf
- File S3.pdf

The copyright of individual parts of the supplement might differ from the article licence.
